# Analysis of Individual Mouse Activity in Group Housed Animals of Different Inbred Strains using a Novel Automated Home Cage Analysis System

**DOI:** 10.3389/fnbeh.2016.00106

**Published:** 2016-06-10

**Authors:** Rasneer S. Bains, Heather L. Cater, Rowland R. Sillito, Agisilaos Chartsias, Duncan Sneddon, Danilo Concas, Piia Keskivali-Bond, Timothy C. Lukins, Sara Wells, Abraham Acevedo Arozena, Patrick M. Nolan, J. Douglas Armstrong

**Affiliations:** ^1^Mary Lyon Centre, Medical Research Council HarwellOxfordshire, UK; ^2^Actual Analytics LtdEdinburgh, UK; ^3^Mammalian Genetics Unit, Medical Research Council HarwellOxfordshire, UK; ^4^School of Informatics, University of EdinburghEdinburgh, UK

**Keywords:** mouse models, mouse behavior, circadian rhythm, strain differences, C57BL/6 mice, inbred mouse strains

## Abstract

Central nervous system disorders such as autism as well as the range of neurodegenerative diseases such as Huntington's disease are commonly investigated using genetically altered mouse models. The current system for characterizing these mice usually involves removing the animals from their home-cage environment and placing them into novel environments where they undergo a battery of tests measuring a range of behavioral and physical phenotypes. These tests are often only conducted for short periods of times in social isolation. However, human manifestations of such disorders are often characterized by multiple phenotypes, presented over long periods of time and leading to significant social impacts. Here, we have developed a system which will allow the automated monitoring of individual mice housed socially in the cage they are reared and housed in, within established social groups and over long periods of time. We demonstrate that the system accurately reports individual locomotor behavior within the group and that the measurements taken can provide unique insights into the effects of genetic background on individual and group behavior not previously recognized.

## Introduction

Basic neuroscience research exploits a wide range of animal models to help dissect structure/function relationships in the brain and the wider nervous system. The majority of biomedical and preclinical research into disease mechanisms and into early drug development relies on the mouse as a surrogate for the human condition.

Rodents used in laboratory research are usually housed in small groups in cages where they eat, sleep, drink, groom, and interact socially. Moreover, animals are often placed in these groups from weaning and are therefore likely to establish high-order social hierarchies (Wang et al., 2014) and behaviors (Shemesh et al., 2013).

The experimental design of many current phenotyping tests relies on the animal being removed from this home-cage environment and placed in an unfamiliar apparatus. Many tests, especially those measuring behaviors (for review see Crawley, 2007), are often laborious, subjective and under the variable influence of an experimenter (Wahlsten et al., 2003); even if the data capture itself can be automated or controlled, the unfamiliar environments and the presence of the experimenter during the test may have an influence on the phenotypic outcome. Mice are social animals in the wild, however, solitary housing is often required for longer-term measures of activity; removing the mouse from its cage-mates and placing them into a novel environment has been shown to affect behavior, general well-being, and metabolism (Bartolomucci et al., 2003; Sun et al., 2014). As an example, social isolation can influence disease progression in a number of neurodegeneration mouse models (Huang et al., 2011).

All these challenges are not new, but with increasing emphasis on reproducibility and robustness of data (Mandillo et al., 2008), the onset of genome editing techniques increasing the number and variety of animal models being generated and the desire to characterize animal models more comprehensively (Perrin, 2014), it is timely to explore new phenotyping paradigms using more naturalistic conditions.

As well as removing bias, non-invasive data recording methods allow cages of mice to be individually monitored for many months with no adverse effect on their welfare. This has the potential to greatly enhance the study of a wide range of neurological diseases by enabling the accurate measurement of progressive behavioral changes in the same animal (e.g., Brooks et al., 2012). Likewise, these systems are well-placed for improving short-term welfare assessment by enabling 24 h monitoring, even in the dark phase where welfare assessment without disturbance to the cage is difficult and subjective (Richardson, 2015).

A range of home-cage analysis systems already exists (for review see Richardson, 2015); all offering unique features, but without the combination of true home-cage monitoring (in the normal rack-mounted cage type the mice are born, reared and constantly housed in, within their established social groups), tracking of each individual and the monitoring of social groups. Most of the existing systems are focused on single animals and/or use essentially bespoke environments (Galsworthy et al., 2005; Morretti et al., 2005; de Visser et al., 2006; Goulding et al., 2008; Freund et al., 2013; Shemesh et al., 2013). For example Intellicage system, measures the activity and reports the number of entries into predetermined activity/testing stations (Vannoni et al., 2014). Though the mice here are group housed, the system itself is not equipped to monitor social groups.

Instead, here we sought to develop a system that was completely compatible with modern high density individually ventilated caging (IVC) systems and capable of collecting spatial data for each individual animal at any given point in time. We aim to automate the collection of a range of behavioral measurements within the home-cage itself in multiple-housed animals. In doing so, we remove the presence of any possible experimenter bias, as well as removing any environmental perturbations whilst maintaining the social grouping within the cage. The system allows for the collection of longitudinal data on individual animals that are housed within their established social groups.

## Methods

### Animals and husbandry

Male mice from three inbred strains—C57BL/6J, C57BL/6NTac, and C3H/HeH, bred at the Mary Lyon Centre, Harwell, were housed in IVC's in groups of three mice per cage (total *n* = 42–45, per strain). The mice were kept under controlled light (light 7 a.m. to 7 p.m., dark 7 p.m. to 7 a.m.), temperature (21 ± 2°C) and humidity (55 ± 10%) conditions. They had free access to water (25 p.p.m. chlorine) and were fed *ad libitum* on a commercial diet (SDS Rat and Mouse No.3 Breeding diet (RM3). All procedures and animal studies were carried out in accordance with the Animals (Scientific Procedures) Act 1986, UK, Amendment Regulations 2012 (SI 4 2012/3039).

Three days prior to recording sessions, the animals were transferred to clean home cages with fresh bedding, nesting material, and a cardboard rodent tunnel as enrichment material, in line with the standard husbandry procedures for IVC cages. When the animals were reared in a different room, their cages after cleaning were placed in an IVC rack in the experimental room for the animals to acclimatize. For each recording, the cages were randomly assigned to an HCA rig. On the first day of recording, each cage was placed onto the ventilation system, within the rig, as would occur during a normal husbandry procedure.

Animal welfare checks were carried out visually twice daily. At the end of the recording period, the home cages were removed from the HCA rigs and returned to their original positions on the IVC racks.

For continuous assessment of activity we selected, at random, six cages of male C57BL/6J, C57BL/6Ntac, and C3H/HeH mice (total *n* = 54) to record using the HCA setup. 31–35 weeks old mice were placed in the rigs and data collected for 7 consecutive days in standard 12 h light/dark (LD) cycles.

### Microchipping

At 12 weeks of age, RFID microchips were injected subcutaneously into the lower left or right quadrant of the abdomen of each mouse. These microchips were contained in standard ISO biocompatible glass capsule (11.5 × 2 mm, PeddyMark Ltd. UK). The procedure was performed on sedated mice (Isoflo, Abbott, UK) after topical application of local anesthetic cream on the injection site prior to the procedure (EMLA Cream 5%, AstraZeneca, UK).

In order to implant the chip, locally anesthetized and sedated mice were placed on their back to allow easy of access to the site of implant, with the snout placed into the gas mask for maintaining sedation. A section of abdominal skin from the lower left quadrant was lifted between the thumb and forefinger. The microchip was inserted using the implant device (a modified syringe) supplied by the RFID manufacturer (PeddyMark Ltd.UK) subcutaneously into this fold of skin (no sutures were required). The mice were removed from the mask and placed into a recovery cage. Once the animals recovered and were mobile again, they were observed for any signs of distress or pain. Once full recovery was confirmed they were placed back into their home cage which was returned to its original position on the IVC rack. The animals were checked after 24 h for any signs of trauma or discomfort and to ensure that the microchips were still in place. The animals were allowed to recover from the microchip procedure for at least 1 week before placing them in the HCA rigs for collecting data.

To determine the long-term effect of microchipping, 64 of the 847 total mice microchipped, including those whose data are reported in the current study, underwent standard necropsy (Scudamore, 2014) at ages ranging from 12 to 21 months.

### Description of rig

**Figure 1:**
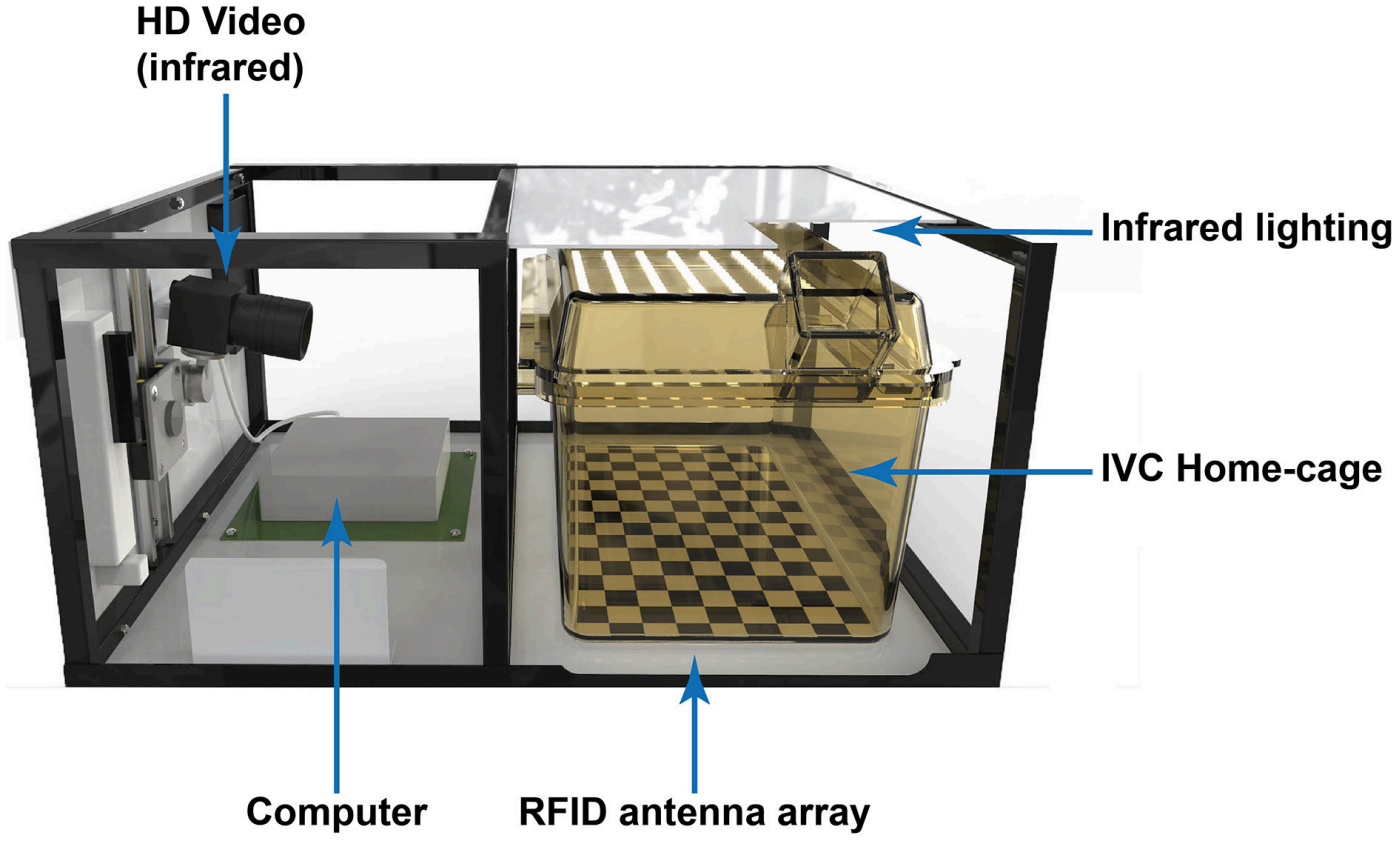
**Illustration of the Home Cage Analysis system with major components highlighted**. The frame shown in the illustration varies according to the rack into which it is installed.

The HCA system (Actual Analytics Ltd, UK), allows one to monitor a cage of mice, and has been designed to fit into two rack spaces of a standard IVC rack (see Figure 1; Single sided seal safe rack, 1284 L holding 56 cages, Techniplast UK Ltd). One half of the rig comprises an RFID reader baseplate with antennae located on predetermined locations. This provides the base for placing the individually ventilated mouse home cage. The other half, fitted within the adjacent rack space, houses an infrared camera, a computer and the appropriate power supplies. The cage sits under a plate affixed to the top of the rig which is fitted with an infrared light source allowing for continuous video capture without compromising the quality of the image.

The size of the electromagnetic field around each antenna is a trade-off between signal strength and spatial resolution. Small fields have better resolution but the field is weaker and therefore the RFID chip needs to be very close to the base. Conversely, increased field strength results in a broader field with lower spatial resolution but the ability to read further away. We selected a range of 20–40 mm read height to allow for the plastic in the home-cage floor, some bedding material, and the tissue of the mouse. To help increase the sensitivity of the system we also injected the RFID chips into the groin of the mouse so they would be nearer the baseplate antennae (see Methods).

To achieve spatial monitoring of location and detect activity, we mount a low profile base-plate that contains a 2D array of 18 RFID antennae (in a 3 × 6 array) directly beneath the home-cage. To achieve sufficient spatial resolution we developed and tested a new high-density and ultra-low profile detector array to track individual position and identity, while still fitting in the tight space tolerance available in modern IVC racks. Each of the antennae in the baseplate is designed to energize a small spatial area within the cage and report the identity of an individually tagged animal within that space. We also added an infrared light source and infrared camera to record (from the side) video footage for validation and, in future, automated behavior recognition (in preparation). A small computer is included to record the data and the system is completed by a frame to match the rack it is installed into and the appropriate power supply units (see Figure 1).

The complete physical system occupies two spaces in a standard IVC mouse rack and holds one standard, unmodified cage (i.e., 50% occupancy in a full rack).

### Data capture

The software package, ActualHCA-Capture (Actual Analytics Ltd, UK) was used to capture readings from the baseplate antennae as well as synchronized video for subsequent validation work. For each recording, the duration of the recording and the length of each recorded segment to be captured could be specified. Typically we used 30 min video segments with a matched series of antenna readings from the baseplate. Once initiated, the recording was allowed to proceed without user interference for the duration of the recording.

The baseplate and video data were amalgamated using ActualHCA Analysis tool v 2.2.2 (Actual Analytics Ltd, UK; from here on referred to as the analysis tool). In order to generate an accurate video overlay, the analysis tool was calibrated to the relevant baseplate coordinates of the specific enclosure. To achieve this, a video of an empty enclosure without a home cage was made and the pixel position of each antenna center on the base-plate grid was mapped into the configuration file.

The data from the baseplate files could also be analyzed separately to provide measurements of the activity of the mice as determined by the readings from the antennae. Unless stated otherwise, for all the experiments described here, activity data was binned into 6 min time bins and is expressed as the total distance traveled in millimeters.

Further visualization of the data was achieved using the data visualization package Tableau Desktop version 9.0 b and custom scripts developed in Python version 2.7 were plotted in Matplotlib version 1.5 (Hunter, 2007). Final figures and images were assembled in Adobe Illustrator and Photoshop.

In order to acquire top-down videos, a rig was removed from the IVC rack, placed on a flat surface and the roof plate removed. The infra-red filter from the camera was also removed and the camera was then suspended vertically from a tripod above the rig and set up such that the entire baseplate was in view. A calibration video of the base plate was then acquired as described above.

A home cage containing three mice was positioned on the baseplate such that the entire cage could be visualized. The nylon lid was removed but the wire bar lid was left in place. A 20 min video of the mice was then acquired. At the end of the experiment the nylon lid was replaced on the home cage and the cage returned to its original position on the IVC rack.

In order to validate the automated overlays, manual annotation was performed on the top-down video files using the program Anvil (v5.1.9, M Kipp). The movement of each individual mouse was tracked manually by clicking on the mouse image on the video every 25 frames (1 s of video). This provided a relative map of the mouse movement over time. As mice can move significantly in 1 s, the videos were subsequently checked at 25 fps to ensure no large movements were missed in the annotation process. The manually annotated mouse coordinates recorded in Anvil were then converted to mm, using a simple projective transformation derived from the calibration pattern present on the surface of the baseplate. This allowed distances traveled by the mice to be measured over the course of the recording, and compared—over segments of recorded footage—to the same distances as estimated from baseplate readings. These manual annotations also allow the accuracy of instantaneous RFID-based location estimates to be measured.

Baseplate and video data for individual animals were recorded continuously in group-housed conditions for periods of up to 7 days. For additional comparison, activity was compared to activity data generated by circadian wheel running analysis. Wheel running activity was performed as outlined in Banks and Nolan (2011). Briefly, C57BL/6J mice (12-13 weeks old) were singly housed in cages containing running wheels. The cages were placed in light controlled chambers for 7 days in a 12-h light dark cycle (100 lux light intensity). Data for the wheel running activity was collected in ClockLab (Actimetrics) and exported as text files and visualized as double plotted raster plots in the data visualization package Tableau Desktop version 9.0 b.

### Statistical analysis

Unless otherwise stated, data were analyzed using One-Way ANOVA followed by *post-hoc* Tukey's test. The analysis was carried out using the Single Measure Parametric Analysis tool of InVivoStat software 3.2.0.0 (Bate and Clarke, 2014) and “multcomp” package in R (Hothorn et al., 2008).

The automatic onset and offset detection of the daily activity rhythm is based on the method described in Chronoshop (Spoelstra, 2010). In brief, the algorithm approximates the rhythm with a sinusoidal signal under the assumption that the rhythm exhibits periodic oscillations. At first, Centre of Gravity (CoG) is calculated, which corresponds to the maximum values of every circadian cycle. Assuming a known period, the onset is defined as the first moment that exceeds the average activity starting from 0.5 cycle before the CoG. This estimation is performed on a smoothed signal to avoid premature onsets. Similarly, the offset of the rhythm is defined as the last moment that exceeds the average activity before the end of the cycle.

The CoG is estimated by the single-component cosinor method, which fits a cosine signal to the locomotor activity data using least squares optimization (Refinetti et al., 2007). The fitted model can be described by the equation:

(1) $$\begin{array}{l} \text{x}\left(\text{t}\right)=\text{M}+\text{Acos}\left(2\pi \text{t}∕\tau +\varphi \right)+\text{e}\left(\text{t}\right) \end{array}$$

where M is the MESOR (Midline Statistic of Rhythm), A is the amplitude, φ is the CoG, τ is the period and e(t) an independent and normally distributed error term with zero mean and unknown variance σ^2^.

## Results

### Description of system

The Home Cage Analysis (HCA) system is entirely built around a normal IVC home-cage designed for a small social group of mice. All the studies here were performed using Techniplast IVC SealSafe Blue line cages.

Radio frequency identity tags (RFID) are already widely used in the field and involve the non-surgical implantation of minute, low-cost RFID asset tags into each animal.

### Microchipping

No obvious adverse reactions to the injected RFID were noted. There were no effects on the welfare of the animals throughout their life time, the body weights were maintained and there were no signs of discomfort or any obvious gait abnormalities observed in any of the animals.

At necropsy, the site of implantation was examined. Fifty seven of the sixty four chips were found to be in place and seven had migrated into the scrotal sac. At necropsy there was no evidence of tissue reddening, thickening or fluid accumulation associated with any of these 64 chips. There was no obvious wound or scar in the abdominal wall of the mice whose chips had migrated into the scrotal sac. One 12 month old C3H/HeH mouse was found to have a small cyst around the end of the implant. Of the seven mice where the chips had migrated into the scrotal sac, three were C57BL/6J, two C57BL/6Ntac, and one each of A/J and H:CD1. One of these C57BL/6Ntac was associated with abnormal tissue findings within the abdominal cavity (kidney and spleen) on the same side as the implant but there was no macroscopic evidence of inflammation around the microchip and therefore of unknown relevance to the RFID chip.

### Top-down validation on group housed animals and results

**Figure 2:**
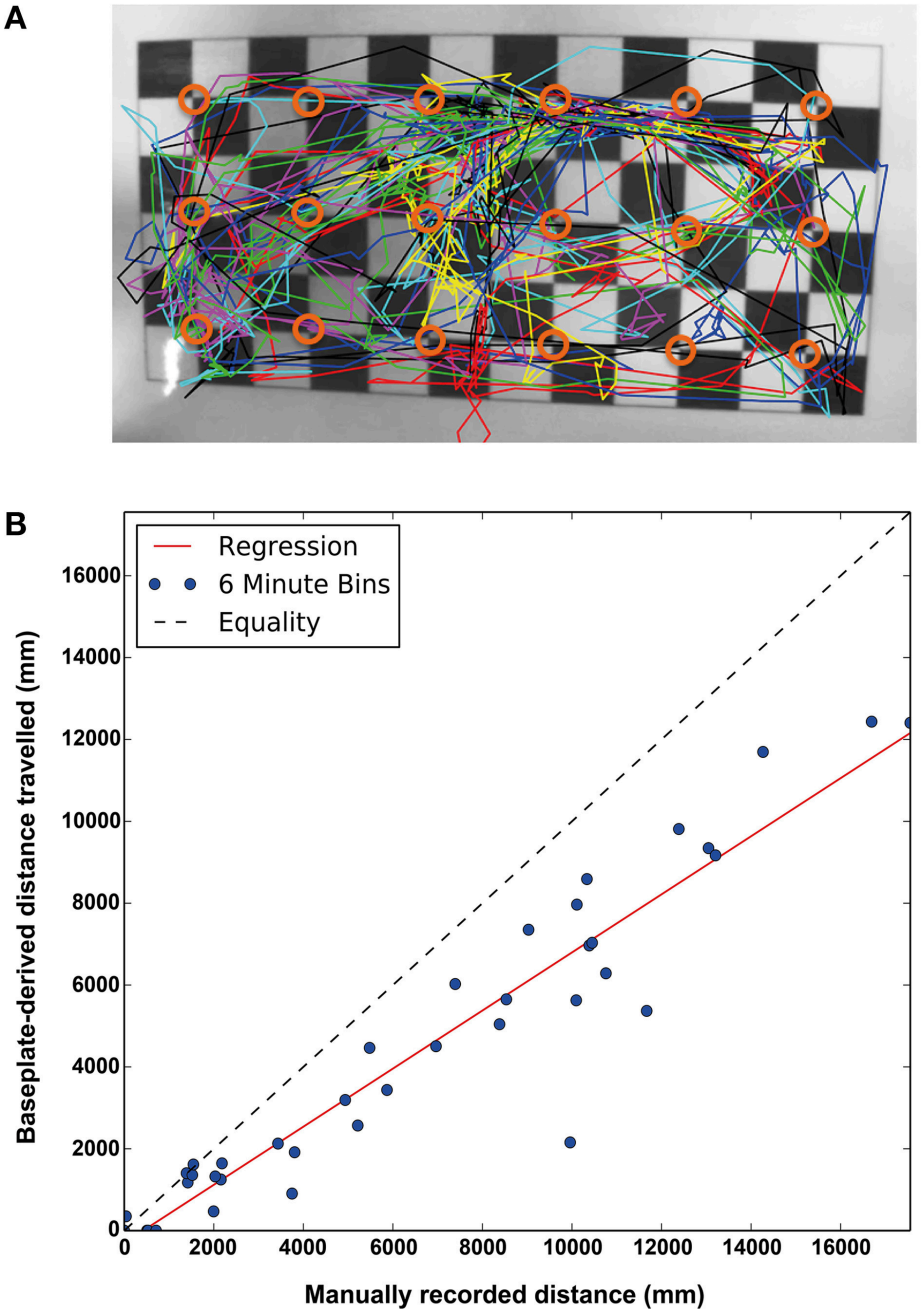
**(A)** Top down view of a baseplate with the manual traces of the animals overlaid. The center point of each RFID antenna is indicated (open orange circle). As the baseplate reads each location as the center of the antenna, the measurements will, on average, underestimate distance moved but with a strong correlation (Spearman's rank coefficient ρ = 0.940, *p* = 6.52 × 10^−19^, *N* = 39) **(B)** The correlation between the actual distance moved and distance estimated by readings from the RFID baseplate. Each point plotted is a single animal recorded and tracked for 6 min. Equality and linear regression lines are shown.

The spatial and temporal resolution of the detection system has physical limitations: Spatially, the array of 3 × 6 RFID detectors means each detection window is ~50 mm in diameter which puts a bound on the specificity of the location returns by a positive read—i.e., each read on a detector says the chip (and hence the mouse) is within the detection field but it cannot describe where exactly within the field. This will cause an expected error in distance between the actual mouse location vs. the position of each antenna that can be predicted mathematically, averaging around 19 mm with a maximum error of 35 mm. This effect is most obvious along the cage boundaries, where an animal situated by the wall will be detected as being in the center of the detection field (see Figure 2A) and the system will systematically under-report the distance moved. As well as estimating this error, we can directly measure it by comparing data obtained with the system to a top view camera (see below), allowing us to develop a correction factor.

Moreover, temporal sampling can also be limited, as each antenna detector is read in sequence, with a temporal resolution of ~8 Hz maximum. The system was designed to skip reads quickly if no chip was present and so the scan rate slows with the number of successful reads. A rate of 2–3 Hz with three animals was usually observed during the studies described here. Further, if an animal is moving quickly across the home-cage during the scan, it can be entirely missed for one or more entire read cycles depending on where the animal is with respect to the active fields. Finally, each antenna detector can only read a single chip ID (presumably the strongest signal) per cycle, therefore, if two or more animals are within the same ~50 mm field, only one animal will be returned per cycle. All these factors combine to mean that the read frequency is always below the physical maximum. Linear interpolation is used to smooth missing reads. The effects of temporal sampling and multiple chips being over a single detector cannot be predicted but we need to be directly measured in observed datasets, as below.

Figure 2A illustrates examples of true subject tracks—tracings from manual annotation using a top-down video against the relative positions of the antennae on the baseplate. For this reason, the baseplate-derived measures of distance traveled systematically underestimate the true distance traveled by the individual mice. We compared the total distance traveled estimated by the baseplate with that measured by the human annotators: each point on the scatter plot (Figure 2B) represents the distance traveled by 1 subject during a 6 min recording session (3 subjects per cage × 13 recordings in total = 39 points). Though these data were collected during the light phase, the amount of disruption caused by removing the cage lids etc. meant that the animals were very active. As discussed above, the estimated distance tends (as shown by the red least-squares regression line) to under-estimate the true distance traveled (as derived from human annotations). Nonetheless, as a means of comparing relative locomotor activity across strains, the baseplate readings provide a useful estimation of activity, showing strong rank correlation with the human annotations (Spearman's rank coefficient ρ = 0.952, *p* = 1.51 × 10^−20^, *N* = 39).

In summary the correlation between estimated distance traveled based on the baseplate alone is strong, allowing us to propose a linear correction factor of 1.4 should an estimate of total distance be critical. However, for studies where there is a paired control, the distance traveled estimated by the baseplate, or even a simple raw count of the transitions between detectors over time, provides a very accurate reflection of the distance moved by individual mice within their home-cage.

### Multiday recordings from laboratory strains

**Figure 3:**
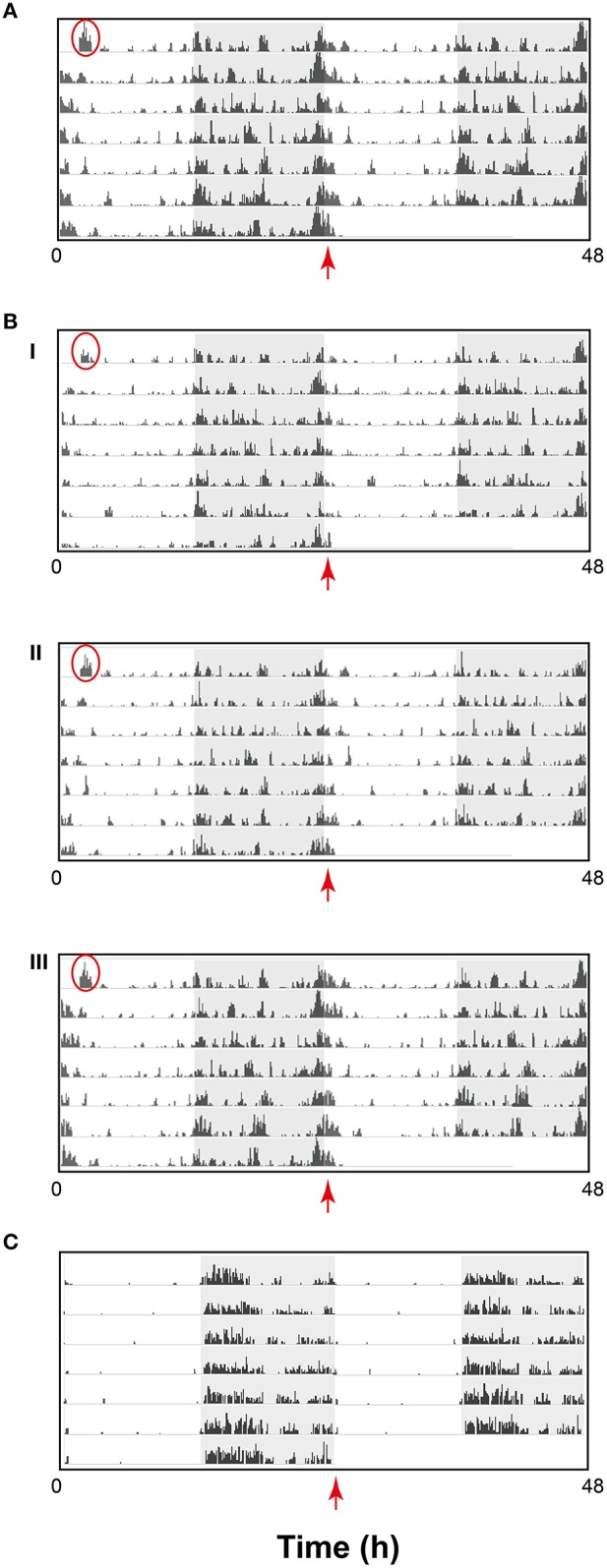
**Activity data from one representative cage of C57BL/6J displayed as a raster plot of the sum of the total distance traveled in millimeter (mm) in 6 min time bins, over 7 consecutive days in standard 12 h light/dark cycles**. The raster plot is double-plotted on a 24 h cycle with the shaded area representing the dark phase. **(A)** Sum of distance traveled by a cage of three animals (scale 20,000 mm) **(B)** Sum of distance traveled by individual animals (i), (ii), and (iii) in the cage represented in A (Scale 10,000 mm) **(C)** Representative example of wheel running in singly-housed C57BL/6J male mouse displayed as a raster plot double-plotted on a 24 h cycle as above where the activity is represented as average counts of wheel rotations in 6 min time bins. Red circles highlight the first bout of activity resulting as a consequence of moving the home cage from its holding IVC rack to the experimental rack. The red arrows highlight activity detected from dawn (ZT0) in the HCA system but not evident using the wheel running-based system.

Having established that activity data could be effectively assessed using the HCA system, we investigated how sensitive and discriminative the system could be over a 7-day recording period. In the first instance, we investigated how an individual's activity pattern fared in the context of the group-housed condition. As an example, activity data from a representative cage of C57BL/6J mice is shown as a double-plotted raster plot (Figures 3A,B). Data is shown as the sum of the distance traveled per 6 min bin by all animals in the cage (Figure 3A) as well as for the distance traveled by each individual within that cage (Figure 3B). The data improve our understanding of how nocturnal animals behave in a home-cage environment. As evidenced in the raster plots, C57BL/6J activity is not entirely confined to the dark phase nor is it consistently high throughout this period, instead showing repetitive patterns of increased or decreased activity over the course of the dark phase. Although, the amount of activity varied amongst individuals, patterns of activity were remarkably similar. Perhaps the most interesting observation is that the most active period for this strain begins just before dawn and is maintained for several hours into the light phase. This is not observed in individually housed mice (Goulding et al., 2008; Loos et al., 2014). The plots also demonstrate how animal behavior can be disturbed by external events. For example, the first bout of activity, encircled in red in each plot, is a consequence of moving the home cage from its holding IVC rack to the experimental rack. This can typically last for up to 60 min after which the animals settle down.

A standard means of testing activity continuously over long periods is to measure wheel-running activity in singly-housed animals. A typical raster plot of wheel running in C57BL/6J mice (Figure 3C) indicates how wheel-running may be misinterpreted as activity. Although the data is not directly comparable to the data in Figure 3B, there are a number of clear differences. In contrast to HCA-based activity, wheel-running activity is predominant during the early part of the dark phase, does not persist through dawn into the light phase and is virtually absent through the rest of the light phase. Moreover, there is clear evidence of pre-dark phase anticipatory activity in C57BL/6J mice assessed using the HCA system, while this is not evident from wheel-running data.

**Figure 4:**
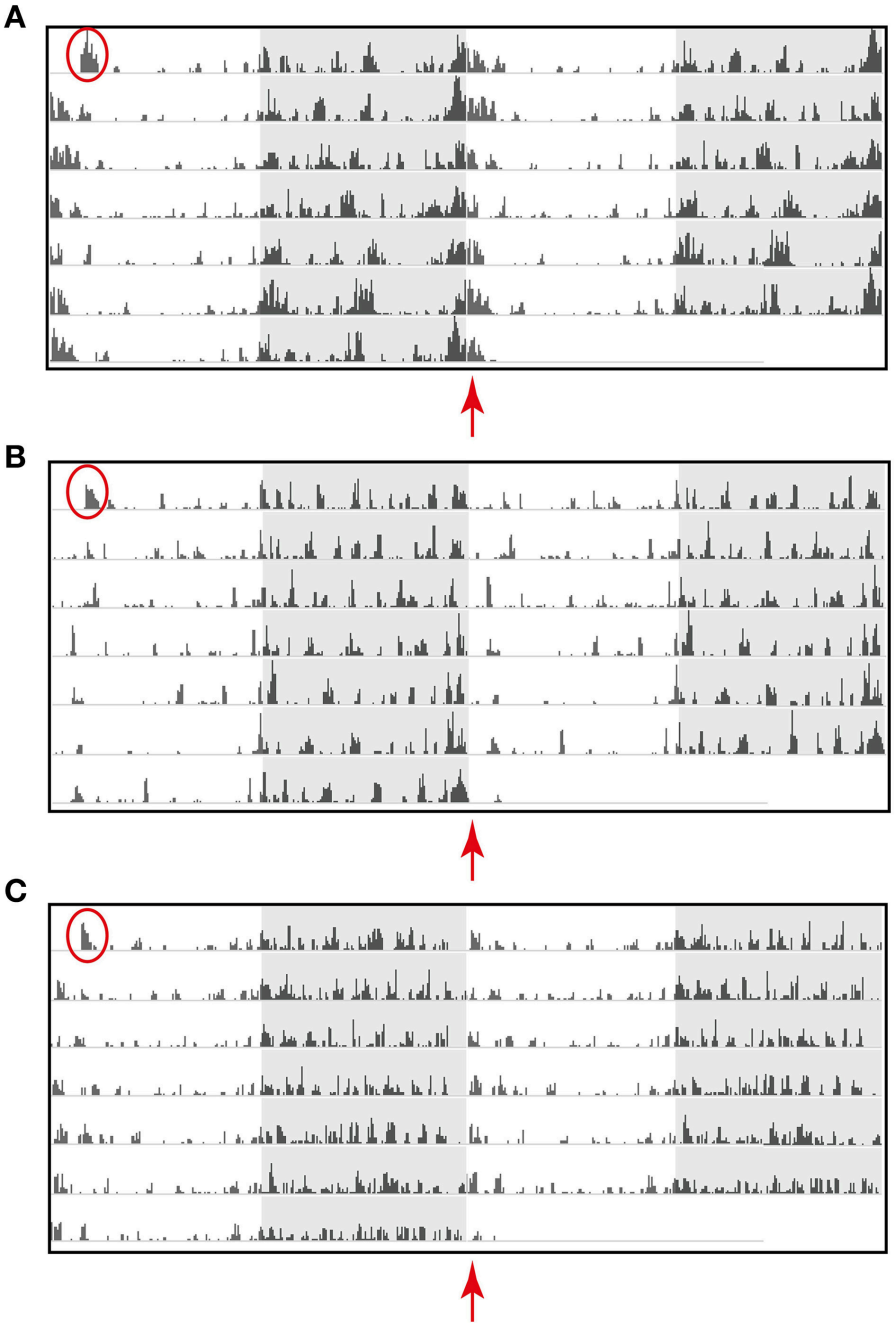
**Activity data for a representative individual mouse from a cage of three (A) C57BL/6J (scale 10,000 mm), (B) C57BL/6Ntac (Scale 6500 mm), and (C) C3H/HeH (scale 6500 mm) displayed a raster plot of the total distance traveled in millimeter (mm) in 6 min time bins, over 7 consecutive days in standard 12 h light/dark cycles**. The raster plot is double-plotted on a 24 h cycle with the shaded area representing the dark phase. Red circles highlight the first bout of activity resulting as a consequence of moving the home cage from its holding IVC rack to the experimental rack. The red arrows highlight strain differences in activity detected from dawn (ZT0) using the HCA system.

Mouse strain differences in amount and patterns of activity were clearly evident using the HCA system (Figure 4). Representative individual raster plots for C57BL/6J, C57BL/6Ntac, and C3H/HeH highlight these differences. Noticeably, C57BL/6J mice (Figure 4A) are the most active compared to C57BL/6Ntac (Figure 4B) and C3H/HeH mice (Figure 4C). Furthermore, there is a distinct pattern of activity during the dark phase in each strain. For example, differences in activities across the dark-light transition are highlighted by the red arrows. C57BL/6J mice show noticeable peaks of activity throughout the night, with extended activity for up to 60 min after lights on (Figure 4A). C57BL/6Ntac mice also show peaks of activity during the dark phase but there is a suppression of activity at the start of the light phase relative to the other two strains (Figure 4B). In contrast, C3H/HeH mice show sustained bouts of intermediate activity, beginning with clear anticipatory activity prior to lights off and continuing into the dark phase. C3H/HeH mice also show a clear reduction in activity toward the end of the dark phase and an additional short bout of activity at the start of the light phase (Figure 4C).

**Figure 5:**
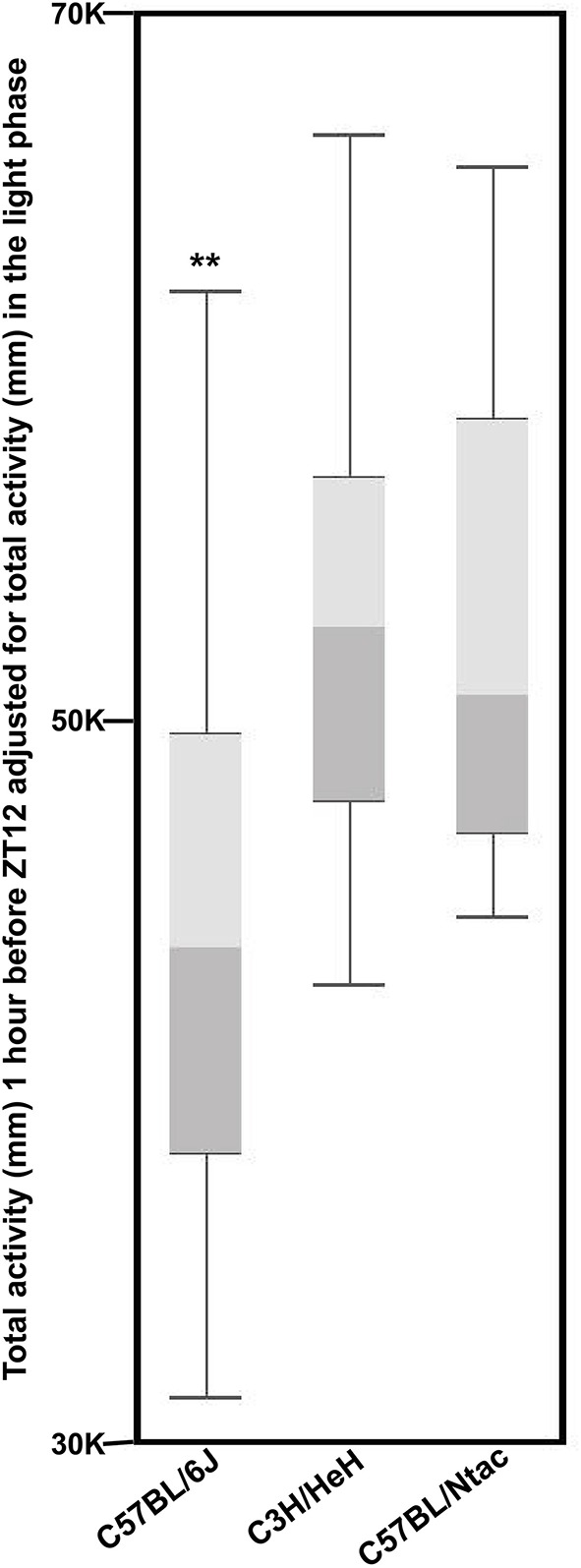
**The sum of activity for three strains (*n* = 54 total) between 18:00 and 19:00 h for 7 days fitted to sum of day time activity for the whole week displayed as a Box and Whisker plot**. Whiskers refer to the data within 1.5 times the interquartile range, the boxes represent the 1st and 3rd quartile around the median. Data were analyzed using Analysis of Variance followed by *Post-hoc* Tukey's test. The results show that: The anticipatory activity of C57BL/6J mice is significantly (^**^p > 0.01) lower than C57BL/6Ntac mice, but no differences were found between C3H/HeH mice and the other two strains.

To quantify if the anticipatory behavior prior to lights off (18:00-19:00) is strain specific, we analyzed the total activity for each animal of each strain 1 h prior to lights off (18:00–19:00). To remove any bias resulting from the different total activities for each strain, we adjusted the total activity for the 18:00–19:00 period to the total activity for each animal during the day (Figure 5; for more details see Supplementary Data Sheets 1,2). We compared the resulting activity for each strain using an analysis of co-variance followed by *post-hoc* Tukey's test. The pairwise results showed that the anticipatory activity of C57BL/6J mice is significantly (*p* > 0.01) lower than C57BL/6Ntac mice, but no differences were found between C3H/HeH mice and the other two strains.

Another noticeable difference in behavior amongst the three strains is the duration of the first bout of activity at the very start of the recording (red circles, Figure 4). As indicated earlier, this is believed to be a consequence of moving the home cage from holding racks to test racking. Analysis of variance for the duration of this first bout of activity revealed a significant difference between strains (*df* = 2, *F* = 7.63, *p* < 0.01). *Post-hoc* Tukey's test revealed that C57BL/6J mice, the most active of the three strains, took significantly longer to settle down (*p* < 0.001 compared to C57BL/6Ntac and *p* < 0.05 compared to C3H/HeH mice). C57BL/6Ntac and C3H/HeH mice show less pronounced activity during this period, which typically lasts for about 60 min.

**Figure 6:**
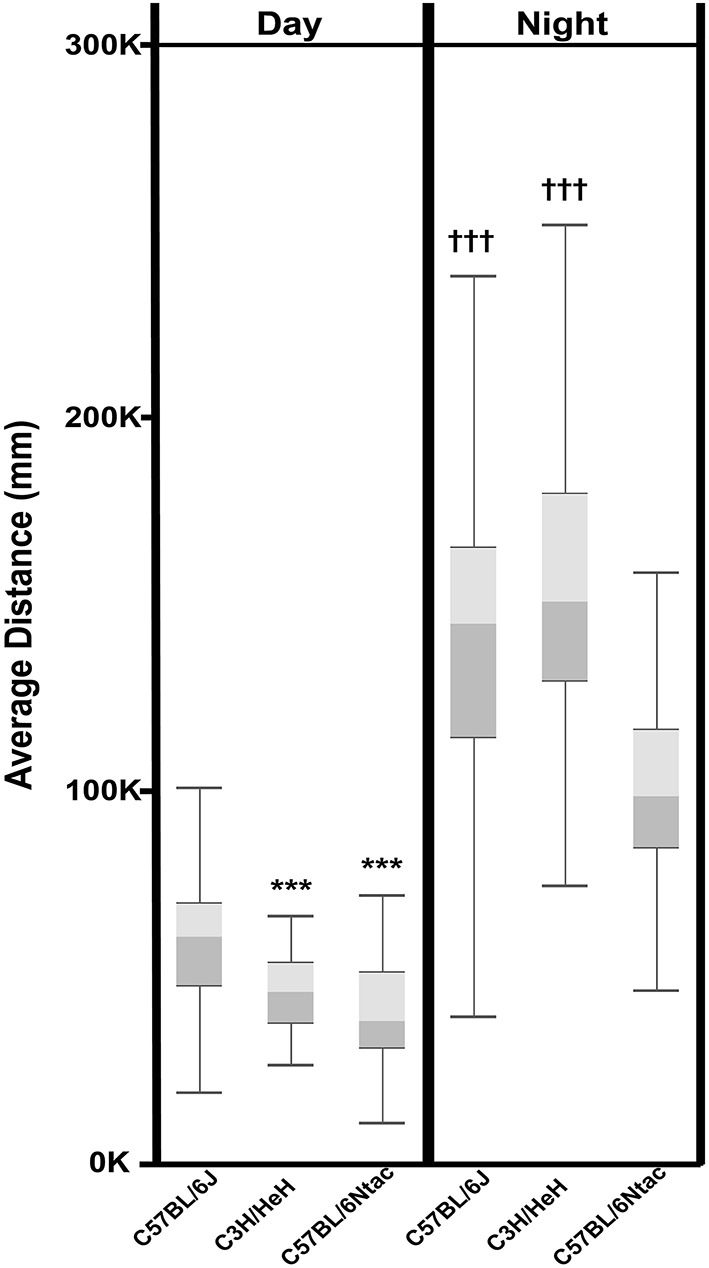
**Total day time and night time activity for three strains displayed as a Box and Whisker plot**. Whiskers refer to the data within 1.5 times the interquartile range, the boxes represent the 1st and 3rd quartile around the median. Data were analyzed using Analysis of Variance followed by *Post-hoc* Tukey's test. The results show that: Total Day Time activity for C3H/HeH and C57BL/6Ntac is significantly lower (^***^p < 0.0001) than that for C57BL/6J. Total Night Time activity for C3H/HeH and C57BL/6J is significantly higher (^†††^p < 0.0001) than that for C57BL/6Ntac (*p* < 0.0001).

Aside from the qualitative differences observed above, we investigated whether we could use the data to extract statistically significant strain differences. To quantitate activity differences between the three inbred strains, data was collected for 72 consecutive h from 13 to 18 weeks old male mice (total *n* = 132). Data collected before the onset of the first dark period was disregarded as this was equated to a period of acclimatization. Data collected after lights on day 3 was also disregarded as this was associated with a period of disturbance when the experiment was stopped. In total, 60 h of data were used to calculate the average distance traveled by each mouse during light and dark phases. As expected, all animals were significantly more active during the dark phase compared to the light phase (Figure 6). In addition, we observed significant strain differences in these activity levels. During the light phase, C57BL/6J mice were significantly (*P* < 0.0001) more active than either C57BL/6Ntac or C3H/HeH mice. During the dark phase however, there was no significant differences between the average activities of C57BL/6J and C3H/HeH (*p* > 0.05) whereas C57BL/6Ntac mice were significantly (*p* < 0.0001) less active than either.

**Figure 7:**
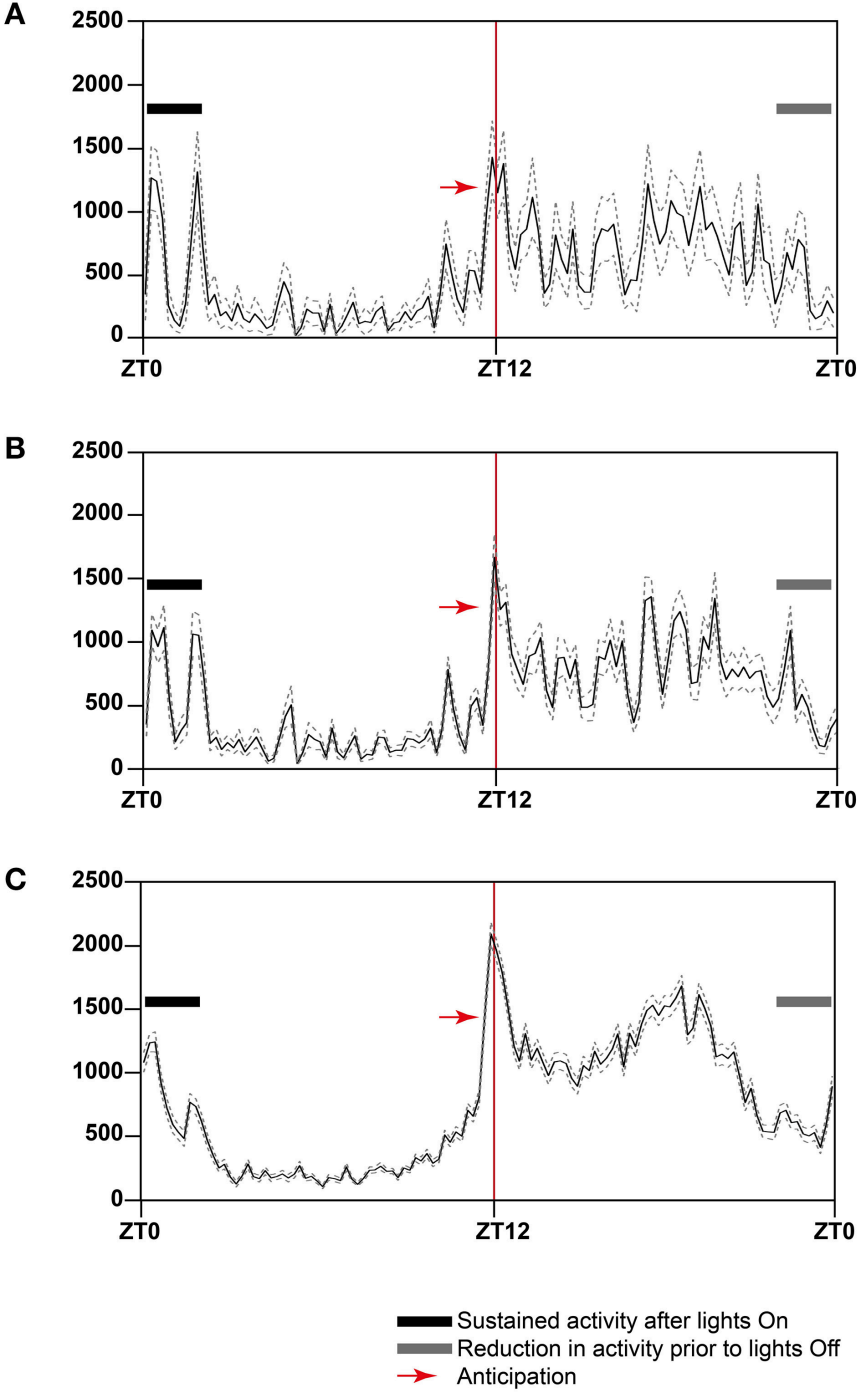
**Twenty-four-hour activity averages over a 7 day period for (A) one C3H/HeH mouse, (B) a cage of three C3H/HeH mice (*n* = 3), and (C) all C3H/HeH mice recorded (*n* = 15)**. The data was plotted in 12 min time bins, represented by the solid line, the dotted lines represent the average ± standard error of mean (SEM). The Y-axis is average total distance measured in mm; the X-axis represents the *zeitgeber* time (ZT), where ZT0 is lights on. The red line at ZT12 indicates where lights are switched off at the beginning of the dark phase. The black bar indicates the period of sustained activity after lights on and the gray bar indicates a period of reduced activity prior to lights on.

**Figure 8:**
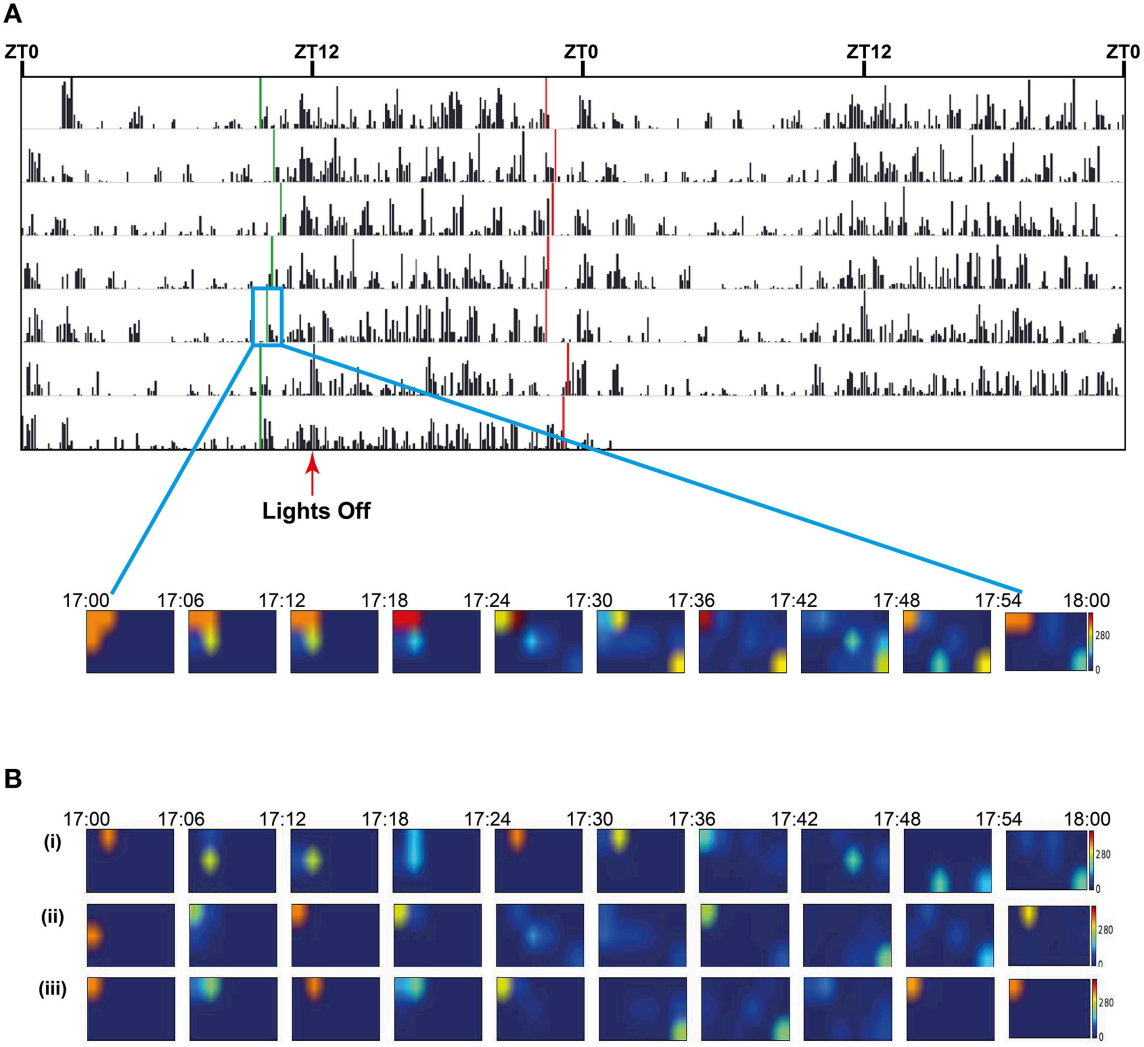
**(A)** Seven day double-plotted actogram for a single animal in a cage of 3, with automatically calculated onset and offset times (green and red vertical bars respectively) indicating activity-related anticipation of the dark and light phases. For the three animals over 7 days in the cage the mean anticipation was 85 min (st dev 36) for lights off and 75 (st dev 38) for lights on. The insert shows a heatmap plot of mean location of the three animals in the cage during the onset period. Prior to onset the individuals are socially clustered in one corner of the cage but, as the time bin representing the activity onset approaches, the mice become more active and mean locations are spread throughout the cage. Each image in the heatmap represents a 6 min bin of locations with the mid-point of the series coinciding with the calculated on-set time (green bar in the box of day 5). **(B)** Heatmap plots of mean location of each of the three animals (i), (ii), and (iii) in the cage during the onset period. Each image in the heatmap represents a 6 min bin of locations with the mid-point of the series coinciding with the calculated on-set time (green bar in the box of day 5). The actogram in **(A)** represents the activity of animal (i).

To highlight consistent patterns of strain activity over a 24 h period, we collected data from 7 day recordings of 31–35 weeks old male C3H/HeH mice, expressing this as the average distance traveled by either: a randomly chosen individual (*n* = 1; Figure 7A), a cage including the individual chosen plus its two cage-mates (*n* = 3; Figure 7B) and the full complement of six cages for the strain (*n* = 18; Figure 7C). There are clear and consistent patterns of strain activity relative to the external light Zeitgeber including a sustained period of elevated activity at the beginning of lights-on, a period of reduced activity toward the end of lights-off and a period of anticipatory activity prior to lights-off. The activity seen prior to lights-off here is true anticipatory activity as the mice are not exposed to a dawn or dusk period where light intensity is gradually reduced/increased. The automated activity onset/offset algorithm accurately predicts these anticipatory episodes in individual mice (Figure 8).

### Animals as a social group

As the system is able to discriminate individuals within a small social group, it also allows us to visualize social interactions within the home-cage group over time. While lights are on, animals are generally huddled together in quietly active clusters. As the time of lights-off approaches, there is a period of anticipation, where the group becomes more active and the distance between animals increases. Mice tend to generally stay further apart during the active dark phase, at least until the anticipation of lights on, when the group clusters back together again. This is shown in a heatmap of positions plotted over time windows around onset of activity at the beginning of the anticipatory period before lights off for day 5 (Figure 8).

## Discussion

Mice are the mammalian organism of choice for the development of neurological disease models. The large numbers of mouse models currently available is already increasing very rapidly due to the advent of novel genome editing technologies such as CRISPR/Cas9, together with the generation of large repositories through large-scale mouse phenotyping programs, including the International Mouse Phenotyping Consortium (IMPC). Thus, there is an urgent need for the development of novel behavioral paradigms to capture and analyze the breadth of mouse models being generated.

Recently a number of technologies using state of the art video recordings combined with infrared beam breaking systems, such as the Photobeam Activity System (San Diego Instruments), have been developed in a bid to automate the scoring process. Such tracking software often only produces one composite parameter and requires housing the animals singly for the duration of the test which may extend to days. In addition to welfare issues related to social isolation (Bibancos et al., 2007), this can result in data that lack consistency as the analysis takes a long time, resulting in smaller sample sizes as well as a reduced number of behaviors analyzed.

The two other popular systems in this area, Phenotyper and Intellicage, have addressed some of these issues with both systems multiplexing a range of tasks into an integrated testing arena and allowing longitudinal studies which reveal strain differences in behavior (e.g., Loos et al., 2014). However, for the most part, testing animals still requires removal from their regular home-cage, usually into social isolation and there is little provision for analyzing multi-participant tasks except for in very controlled situations and these are often focused on pairwise interactions (Morretti et al., 2005; Silverman et al., 2010). Systems capable of analyzing group interactions in three or more mice have been developed (Shemesh et al., 2013). These rely on ultraviolet tracking of labeled mice in the dark phase, although such system address the above mentioned concerns they are not capable of recording data in the light phase.

To our knowledge, this is the first system that is able to distinguish and capture the basal motor activity of multiple-housed mice in normal home cages over long periods of time.

Home-cage systems such as this one require no animal handling, and therefore lead to improvements on animal welfare. This approach does require RFID tagging of the animals which is routine in many facilities and is a minor procedure. We did observe a low frequency of chip migration but found no evidence for adverse effects on the animals concerned. Given the proximity of the site of implantation to the inguinal region, together with the fact that in rodents the inguinal canal remains open throughout their life (Lewis et al., 2012), this is the most likely route of migration. Migration of subcutaneous microchips through normal muscle movement is not uncommon.

In addition, unobtrusive, longitudinal monitoring of group housed animals is particularly desirable for the analysis of progressive motor abnormalities, such as those in neurodegeneration mouse models, as it allows for basal motor activity to be collected at different time-points from the same mice while the disease progresses, without the need for any motor testing.

Moreover, as data is collected from multiple mice in their home-cage, it also potentially allows for the analysis of social interactions within the cage, as well as the automated analysis of home cage behaviors such as drinking, eating or climbing, although these are elements that require further integration with the video feed that we are currently developing.

We have carefully validated the approach by comparing the distance obtained from the baseplate reads of the RFID-tagged mice with various video feeds annotated manually. The correlation between the manually annotated videos and the automatically collected baseplate reads is remarkable. However accuracy does need to be factored in when actual distance moved is important (rather than relative activity between animals, cages or strains) and based on the data described here we can estimate a correction factor of 1.4x is appropriate.

As a proof of principle, we have used the system to capture the basal motor activity of three commonly used inbred strains of mice. As expected, animals were significantly more active during the dark phase compared to the light phase. However, evident bouts of activity were recorded during the light phase for all three strains. This is in contrast to reports where running wheels are used to estimate motor activity, as during the light phase the wheel-running activity is negligible. One of the reasons for this observation may be the difference in light intensity for the two set ups. While the wheel running chambers are maintained under 100 lux light intensity, the HCAs use the same amount of light as a normal IVC on the rack (35–65 lux). In contrast to our system, on free wheel running systems mice are required to be singly housed to be able to estimate their motor activity. Moreover, they do not measure baseline activity, but rather an elective action that could be influenced by many other factors, including motivation. Thus, wheel-running activity is simply measuring a different behavioral output.

The analysis of circadian activity for 7 whole days (and nights) exemplifies the potential of the system. We are able to distinguish, and quantify for statistical analysis, the anticipatory behaviors for all three strains, as their activity increases just before lights-off and decreases just before lights-on. This is not due to light fading at dusk or dawn, as lights are on and off abruptly without warning. Such anticipatory behavior has been observed previously as duration of activity (Nishi et al., 2010; Loos et al., 2014), but is a feature of circadian biology that is not currently captured on free wheel-running systems. It remains to be determined whether such anticipatory activity in a 12:12 h light:dark cycle varies according to the internal circadian period (tau) of the individual and, indeed, whether this can be modified by the social context in the home-cage.

Overall, this novel analysis system will enhance our understanding of how mice behave in their original home-cage. Here we have extensively validated the system, using it initially to study the home-cage activity of commonly used inbred lines. As the system allows for the continuous recording and analysis of baseline activity without experimenter intervention, it will be a powerful new tool to study activities and social interactions in a spectrum of neurological and behavioral mouse models. It will be particularly useful in the investigation of models of progressive motor impairment, such as neurodegenerative conditions, and conditions where social interactions are impaired, such as autism spectrum disorders. The integration of the activity data presented here with the automated analysis of behaviors from the video output that we are currently developing will make this system even more versatile for the capture and automated analysis of complex behaviors from undisturbed mice reared in their home cage.

## Authors contributions

RB Experimental procedures including top down annotation, data analysis, manuscript preparation. HC Study design and manuscript preparation. RS Data visualization (top down study), analysis, and statistics. AC Data analysis for activity and circadian/onset analysis. DS Data collection, bioinformatics, and statistical analysis. DC Regression/statistical analysis. PK Experimental procedures and technical assistance with home-cage equipment. TL System design and implementation. SW Study design including animal procedures and manuscript preparation. AA Study design, activity data analysis and manuscript preparation. PN Study design, circadian and activity data analysis, and manuscript preparation. JA Study design, system design, manuscript writing.

## Funding

The research described in this manuscript was supported by the Crack-It initiative from the National Centre for the Replacement, Refinement and Reduction of Animals in Research (UK) award number NC/C012201/1. This work was additionally supported in part by funding from the Medical Research Council (UK) to the Mary Lyon Centre, Dr. PN (MC_U142684173), Dr. AA (MC_UP_A390_1106), and Dr. A. Mallon (MC_U142684171).

### Conflict of interest statement

The authors RS, AC, TL, and JA were/are employed by or were shareholders in Actual Analytics Ltd at the time the research was performed and therefore declare a competing financial interest. Actual HCA is commercially available from Actual Analytics Ltd.
