# Supplementary material for: Analysis of Individual Mouse Activity in Group Housed Animals of Different Inbred Strains using a Novel Automated Home Cage Analysis System
Source: Front Behav Neurosci. 2016 Jun 10;10:106. doi: 10.3389/fnbeh.2016.00106 (PMC4901040; doi:10.3389/fnbeh.2016.00106)
Supplement: Supplementary file 2 [file DataSheet2.DOCX]

**Bains et al, Supplementary Material**

**Statistical model for anticipation data analysis:**

The sum of the distance for each animal for 1hour prior to lights off (18:00-19:00) for the whole week was regressed against the sum of the daytime (07:00-19:00) distance for that animal for the whole week. An ANCOVA model, built using the R package ‘car’ (1, 2), was used:

lm(Weekly.1h.sum~Strain+Weekly.sum)

The model validity was tested against the heteroscedasticity and the normality criteria according to the description provided by Crawley, 2005 (4).

The strain statistical differences were evaluated by a multiple comparison test with Tukey contrasts on the means using the R package ‘multcomp’ (3).

1. R Core Team (2014). R: A language and environment for statistical computing. R Foundation for Statistical Computing, Vienna, Austria. URL <http://www.R-project.org/>
2. Fox J. and Weisberg S. (2011). *An {R} Companion to Applied Regression*. Second Edition. Thousand Oaks CA: Sage. URL: <http://socserv.socsci.mcmaster.ca/jfox/Books/Companion>.
3. Hothorn T., Bretz F. and Westfall P. (2008). Simultaneous Inference in General Parametric Models. *Biometrical Journal.* **50**:3. 346-363.
4. Crawley M. J. (2005). *Statistics, An introduction using R.* John Wiley & Sons, chapter 11, pp. 200-201, ISBN 0-470-02298-1
